# Supplementary material for: Performance Testing of Bacillus cereus Chromogenic Agar Media for Improved Detection in Milk and Other Food Samples
Source: Foods. 2022 Jan 21;11(3):288. doi: 10.3390/foods11030288 (PMC8834558; doi:10.3390/foods11030288)
Supplement: Supplementary file 1 [file foods-11-00288-s001.zip › foods-1536979-supplementary.pdf]

Supplementary Table S1. Media specifications according to the respective manufacturer's descriptions.

**Agar**		Mannitol egg yolk polymyxin (MYP) agar	ChromoSelect *Bacillus* agar	Brilliance™ *B. cereus* agar	CHROMagar™ *B. cereus*	BACARA® agar
**Manufacturer**		Thermo Fisher Scientific Inc., Oxoid, Waltham, USA	Merck KgaA, Darmstadt, Germany	Thermo Fisher Scientific Inc., Oxoid, Waltham, USA	CHROMagar, Paris, France	bioMérieux, Marcy-l'Étoile, France
**Composition**						
Agar	g/L	12.0	15.0	13.0	15.0	18.0
Meat extract	g/L	1.0	1.0			
Peptic digest of animal tissue	g/L			10.0		10.0
Peptone and yeast extract	g/L				8.0	
Yeast extract	g/L			4.0		4.0
Mannitol	g/L	10.0	10.0			
Sodium chloride	g/L	10.0	10.0		10.0	4.0
Sodium pyruvate	g/L			10.0		
Di-sodium hydrogen phosphate	g/L			2.52		
Phosphate-buffer	g/L					10.0
Potassium di-hydrogen phosphate	g/L			0.28		
Phenol red	g/L	0.025	0.025			
Egg yolk emulsion	mL/L	100				
Phospholipids	n.s.				n.s.	n.s.
Chromogenic mix (X-glucopyranoside)	g/L		3.2	1.2	0.3	1.2
Chromogenic substrate	g/L					0.05
Selective mix	g/L				3.0	
Antibiotic mix	g/L					0.26
Polymyxin B	mg/L, IU/L	100,000 IU	10.0	106,000 IU		
Bacitracin	mg/L		10.0			
Trimethoprim	mg/L			10.0		
pH (25 °C)		7.2 ± 0.2	7.1 ± 0.2	7.2 ± 0.2	6.8 ± 0.2	7.2 ± 0.2
Reactions tested		Mannitol fermentation, phospholipase C (PLC) activity	Mannitol fermentation, β-D-glucosidase activity	β-D-glucosidase activity	β-D-glucosidase activity, phospholipase C (PLC) activity	Enzymatic cleavage of the chromogenic substrate, phospholipase C (PLC) activity
Typical morphology		Bright pink colonies surrounded by a zone of egg yolk precipitation ("halo")	Light blue, large, flat colonies	Blue/green colonies (white if strain is β-D-glucosidase negative)	Blue colonies with white opacification halo	Pink/orange with opacification halo
Incubation conditions (aerobically)		30 °C for 18–48 h	30 °C for 24–48 h	37 °C for 24 h	30 °C for 18–24 h	37 °C for 24 h

Sources: https://www.sigmaaldrich.com; http://www.oxoid.com; https://www.chromagar.com/; https://www.biomerieux-usa.com/sites/subsidiary_us/files/bacara_sales_flyer.pdf. Abbreviations: n.s. — not specified, the grey color indicates absent components in the corresponding agar formulation.

Supplementary Table S2. Inclusivity test strains (*n* = 110).

ID	Species	Strain	Category	Source	*panC*	Tox
BCG 4	*Bacillus pseudomycoides*	WS 3119	Soil	Soil	I	C
BC 1	*Bacillus cereus*	C/1	Milk & dairy products	Pasteurized milk, retail	II	F
BC 5	*Bacillus cereus*	F2404B/79	Diarrheal outbreak	Unknown	II	F
BC 45	*Bacillus cereus*	K195	Milk & dairy products	Pasteurized milk	II	C
BC 50	*Bacillus cereus*	K341	Milk & dairy products	Pasteurized milk	II	F
BC 93	*Bacillus cereus*	EFBC 32	Mushrooms	Dried black mushrooms	II	F
BC 122	*Bacillus cereus*	K194	Milk & dairy products	Pasteurized milk	II	C
BC 126	*Bacillus cereus*	K620	Milk & dairy products	Raw milk	II	C
BC 128	*Bacillus cereus*	K781	Milk & dairy products	Pasteurized milk	II	C
BC 2	*Bacillus cereus*	F3003/73	Diarrheal outbreak	Unknown	III	F
BC 6	*Bacillus cereus*	F3350/87	Emetic outbreak	Unknown	III	E
BC 7	*Bacillus thuringiensis*	F4429/71	Diarrheal outbreak	Vanilla pudding	III	D
BC 8	*Bacillus cereus*	MHI 87	Emetic outbreak	Baby food	III	E
BC 9	*Bacillus cereus*	MHI 135	Emetic outbreak	Baby food	III	E
BC 10	*Bacillus cereus*	MHI 280	Emetic outbreak	Unknown	III	E
BC 11	*Bacillus cereus*	MHI 297	Emetic outbreak	Unknown	III	E
BC 12	*Bacillus cereus*	NVH0861-00	Diarrheal outbreak	Soft ice	III	F
BC 13	*Bacillus cereus*	RIVM-BC51	Emetic outbreak	Rice dish	III	E
BC 24	*Bacillus cereus*	Numico	Milk & dairy products	Milk powder	III	F
BC 58	*Bacillus thuringiensis*	EFBC 1	Milk & dairy products	Feta	III	D
BC 60	*Bacillus thuringiensis*	EFBC 4	Milk & dairy products	Feta	III	G
BC 62	*Bacillus cereus*	EFBC 8	Spices	Orange pepper	III	A
BC 66	*Bacillus cereus*	EFBC 5	Milk & dairy products	Feta	III	D
BC 67	*Bacillus thuringiensis*	EFBC 6	Milk & dairy products	Feta	III	D
BC 68	*Bacillus thuringiensis*	EFBC 10	Spices	Orange pepper	III	F
BC 71	*Bacillus cereus*	EFBC 15	Fruits & vegetables	Water pennywort	III	D
BC 72	*Bacillus thuringiensis*	EFBC 16	Fruits & vegetables	Chinese water spinach	III	F
BC 73	*Bacillus thuringiensis*	EFBC 17	Fish & fishery products	Dried crayfish	III	F
BC 75	*Bacillus thuringiensis*	EFBC 19	Spices	Dried pepper soup	III	F
BC 76	*Bacillus thuringiensis*	EFBC 20	Nuts, nut products & seeds	Ground egusi	III	D
BC 77	*Bacillus thuringiensis*	EFBC 21	Cereals & pastries	Rice flakes	III	F
BC 79	*Bacillus cereus*	EFBC 42	Mushrooms	Dried black mushrooms	III	D
BC 81	*Bacillus thuringiensis*	EFBC 45	Fish & fishery products	Dried salted anchovies	III	F
BC 83	*Bacillus cereus*	EFBC 39	Tea	Bitter melon tea	III	A
BC 84	*Bacillus cereus*	EFBC 38	Tea	Bitter melon tea	III	A
BC 86	*Bacillus cereus*	EFBC 41	Nuts, nut products & seeds	Ground egusi	III	A
BC 87	*Bacillus cereus*	EFBC 26	Fish & fishery products	Dried salted anchovies	III	D
BC 89	*Bacillus cereus*	EFBC 25	Fish & fishery products	Dried shrimps	III	D
BC 91	*Bacillus thuringiensis*	EFBC 30	Mushrooms	Shiitake mushrooms	III	D
BC 94	*Bacillus cereus*	EFBC 36	Nuts, nut products & seeds	Saté peanut sauce	III	D
BC 127	*Bacillus thuringiensis*	K760	Milk & dairy products	Pasteurized milk	III	C
BCG 10	*Bacillus cereus*	EFBC 18	Mushrooms	Dried black mushrooms	III	A
BCG 62	*Bacillus cereus*	EFBG 56	Mushrooms	Dried black mushrooms	III	D
BC 15	*Bacillus cereus*	WSBC 10028	Milk & dairy products	Pasteurized milk	IV	A
BC 17	*Bacillus cereus*	WSBC 10201	Milk & dairy products	Pasteurized milk	IV	A

BC 22	*Bacillus cereus*	ATCC 11778	Unknown	Unknown	IV	D
BC 23	*Bacillus cereus*	DSM 31	Unknown	Unknown	IV	A
BC 28	*Bacillus thuringiensis*	NCTC 7464	Unknown	Unknown	IV	A
BC 29	*Bacillus cereus*	K3	Milk & dairy products	Raw milk	IV	A
BC 30	*Bacillus cereus*	K7	Milk & dairy products	Raw milk	IV	A
BC 31	*Bacillus cereus*	K14	Milk & dairy products	Raw milk	IV	A
BC 32	*Bacillus cereus*	K20	Milk & dairy products	Raw milk	IV	C
BC 33	*Bacillus cereus*	K27	Milk & dairy products	Raw milk	IV	C
BC 44	*Bacillus thuringiensis*	K193	Milk & dairy products	Raw milk	IV	A
BC 61	*Bacillus thuringiensis*	EFBC 7	Spices	Saatar spice	IV	A
BC 63	*Bacillus thuringiensis*	EFBC 9	Spices	Orange pepper	IV	D
BC 64	*Bacillus thuringiensis*	EFBC 12	Fruits & vegetables	Dried apricots	IV	C
BC 65	*Bacillus thuringiensis*	F1	Delicacies	Spread	IV	D
BC 69	*Bacillus thuringiensis*	EFBC 11	Fruits & vegetables	Dried apricots	IV	C
BC 70	*Bacillus cereus*	EFBC 14	Fruits & vegetables	Okra	IV	A
BC 78	*Bacillus thuringiensis*	EFBC 22	Cereals & pastries	Rice flakes	IV	A
BC 82	*Bacillus thuringiensis*	EFBC 37	Tea	Vietnamese green tea	IV	A
BC 85	*Bacillus cereus*	EFBC 40	Spices	Fish Hara seasoning mix	IV	A
BC 88	*Bacillus thuringiensis*	EFBC 35	Tea	Bitter melon tea	IV	A
BC 90	*Bacillus thuringiensis*	EFBC 29	Mushrooms	Shiitake mushrooms	IV	A
BC 92	*Bacillus cereus*	EFBC 31	Mushrooms	Dried white mushrooms	IV	A
BC 95	*Bacillus thuringiensis*	EFBC 33	Tea	Vietnamese green tea	IV	A
BC 100	*Bacillus cereus*	K12	Milk & dairy products	Raw milk	IV	A
BC 101	*Bacillus cereus*	K16	Milk & dairy products	Raw milk	IV	A
BC 102	*Bacillus cereus*	K18	Milk & dairy products	Raw milk	IV	A
BC 103	*Bacillus cereus*	K31	Milk & dairy products	Raw milk	IV	A
BC 104	*Bacillus cereus*	K22	Milk & dairy products	Raw milk	IV	D
BC 105	*Bacillus cereus*	K2	Milk & dairy products	Raw milk	IV	D
BC 106	*Bacillus cereus*	K4	Milk & dairy products	Raw milk	IV	A
BCG 12	*Bacillus cereus*	EFBG 41	Tea	Bitter melon tea	IV	A
BCG 63	*Bacillus thuringiensis*	68-1	Mushrooms	Shiitake mushrooms	IV	A
BC 25	*Bacillus toyonensis*	S4-1	Fruits & vegetables	Prewashed salad	V	C
BC 26	*Bacillus thuringiensis*	S26-1	Fruits & vegetables	Prewashed salad	V	C
BC 38	*Bacillus toyonensis*	K49	Milk & dairy products	Raw milk	V	C
BC 80	*Bacillus thuringiensis*	EFBC 44	Fresh herbs	Coriander	V	C
BC 118	*Bacillus toyonensis*	K50	Milk & dairy products	Raw milk	V	C
BC 129	*Bacillus thuringiensis*	890	Milk & dairy products	Pasteurized milk	V	F
BC 130	*Bacillus toyonensis*	K923	Milk & dairy products	Pasteurized milk	V	C
BCG 6	*Bacillus toyonensis*	S16-1	Fruits & vegetables	Prewashed salad	V	C
BCG 7	*Bacillus thuringiensis*	S21-1	Fruits & vegetables	Prewashed salad	V	F
BCG 8	*Bacillus toyonensis*	S36-1	Fruits & vegetables	Prewashed salad	V	C
BCG 11	*Bacillus toyonensis*	EFBC 43	Mushrooms	Mu-Err mushrooms	V	D
BCG 22	*Bacillus thuringiensis*	CCM 1462	Unknown	Unknown	V	D
BC 14	*Bacillus mycoides*	SDA MA57	Milk & dairy products	Raw milk	VI	C
BC 19	*Bacillus mycoides*	WSBC 10207	Milk & dairy products	Pasteurized milk	VI	F
BC 20	*Bacillus mycoides*	WSBC 10377	Milk & dairy products	Raw milk	VI	C
BC 21	*Bacillus mycoides*	WSBC 10208	Milk & dairy products	Pasteurized milk	VI	C
BC 27	*Bacillus mycoides*	S73-2	Fruits & vegetables	Prewashed salad	VI	C
BC 34	*Bacillus mycoides*	K32	Milk & dairy products	Raw milk	VI	A

BC 35	*Bacillus mycoides*	K41	Milk & dairy products	Raw milk	VI	C
BC 36	*Bacillus weihenstephanensis*	K43	Milk & dairy products	Raw milk	VI	C
BC 37	*Bacillus mycoides*	K45	Milk & dairy products	Raw milk	VI	C
BC 39	*Bacillus mycoides*	K57	Milk & dairy products	Raw milk	VI	C
BC 108	*Bacillus mycoides*	K58	Milk & dairy products	Raw milk	VI	C
BC 110	*Bacillus mycoides*	K38	Milk & dairy products	Raw milk	VI	C
BC 111	*Bacillus mycoides*	K34	Milk & dairy products	Raw milk	VI	C
BC 112	*Bacillus mycoides*	K37	Milk & dairy products	Raw milk	VI	C
BC 113	*Bacillus mycoides*	K42	Milk & dairy products	Raw milk	VI	C
BC 116	*Bacillus mycoides*	K46	Milk & dairy products	Raw milk	VI	C
BC 117	*Bacillus mycoides*	K48	Milk & dairy products	Raw milk	VI	C
BCG 1	*Bacillus mycoides*	KBAB4	Soil	Soil	VI	C
BCG 2	*Bacillus mycoides*	MC67	Soil	Soil	VI	C
BCG 3	*Bacillus weihenstephanensis*	WSBC 10204	Milk & dairy products	Raw milk	VI	C
BCG 9	*Bacillus mycoides*	S49-2	Fruits & vegetables	Prewashed salad	VI	C
BCG 32	*Bacillus mycoides*	S13-2	Fruits & vegetables	Prewashed salad	VI	F

Abbreviations: *panC* — *panC* group; Tox — toxin gene profile; POS — positive reaction; NEG — negative reaction; A — toxin profile A (*nhe*+/*hbl*+/*cytK*+); C — toxin profile C (*nhe*+/*hbl*+); D — toxin profile D (*nhe*+/*cytK*+); E — toxin profile E (*nhe*+/*ces*+); F — toxin profile F (*nhe*+); II — *panC* group II (highly cytotoxic, growth ≥ 7°C); III — *panC* group III (highly cytotoxic, growth ≥ 15°C); IV — *panC* group IV (highly cytotoxic, growth ≥ 10°C); V — *panC* group V (low cytotoxic, growth ≥ 8°C); VI — *panC* group VI (not or little cytotoxic; growth ≥ 5°C).

Supplementary Table S3. Exclusivity test strains (n = 110).

ID	Strain	Category	Source	Species
BG 13	10; V/05	Fish & fishery products	Smoked salmon	*Bacillus circulans*
BG 14	9; V/06	Fish & fishery products	Smoked salmon	*Bacillus circulans*
BG 15	BS	Vegetarian meat substitutes	Vegetarian sausage	*Bacillus circulans*
BG 16	5 V/05	Fish & fishery products	Smoked salmon	*Bacillus circulans*
BG 18	BS	Vegetarian meat substitutes	Vegetarian sausage	*Bacillus clausii*
BG 19	DSM 9	Environment	Soil	*Bacillus lentus*
BG 20	CCM 2145	Unknown	Unknown	*Bacillus licheniformis*
BG 23	CCM 2144	Unknown	Unknown	*Bacillus pumilus*
BG 24	BS	Vegetarian meat substitutes	Vegetarian sausage	*Bacillus pumilus*
BG 26	BF47=25	Meat & meat products	Raw sausage	*Bacillus pumilus*
BG 27	AGES CCHX3	Milk & dairy products	ESL-milk	*Bacillus safensis*
BG 28	BS	Vegetarian meat substitutes	Vegetarian sausage	*Bacillus stratosphericus*
BG 29	BS	Vegetarian meat substitutes	Vegetarian sausage	*Bacillus subtilis*
BG 30	NCTC 10400	Unknown	Unknown	*Bacillus subtilis*
BG 31	2; V/05	Fish & fishery products	Smoked salmon	*Bacillus oleronius*
BG 33	BS	Vegetarian meat substitutes	Vegetarian sausage	*Oceanobacillus polygoni*
BG 34	JB	Milk & dairy products	Raw milk	*Paenibacillus peoriae*
BG 35	DSM 36	Unknown	Unknown	*Paenibacillus polymyxa*
BG 36	9-2	Milk & dairy products	Goat cheese	*Lysinibacillus boronitolerans*
BG 37	13-1	Spices	Curry powder	*Brevibacillus halotolerans*
BG 38	13-1	Spices	Curry powder	*Bacillus amyloliquefaciens*
BG 39	14-1	Spices	Paprika flakes	*Bacillus amyloliquefaciens*
BG 40	14-2	Spices	Paprika flakes	*Bacillus amyloliquefaciens*
BG 41	17-1	Spices	Kebab spice	*Bacillus amyloliquefaciens*
BG 43	19-1	Fruits & vegetables	Apricots	*Brevibacillus laterosporus*
BG 44	E1	Delicacies	Spread	*Bacillus subtilis*
BG 45	E3	Delicacies	Spread	*Bacillus subtilis*
BG 46	F2	Delicacies	Spread	*Bacillus safensis*
BG 47	3-1	Delicacies	Stuffed vine leaves	*Bacillus safensis*
BG 48	4-1	Delicacies	Black olives	*Bacillus paralicheniformis*
BG 49	6-1	Delicacies	Aubergine salad	*Bacillus amyloliquefaciens*
BG 50	11-1	Meat & meat products	Brawn	*Bacillus safensis*
BG 51	13-1	Spices	Curry powder	*Bacillus tequilensis*
BG 52	14-1	Spices	Paprika flakes	*Bacillus amyloliquefaciens*
BG 53	16-1	Spices	Saatar spice	*Bacillus tequilensis*
BG 54	16-1	Spices	Saatar spice	*Bacillus safensis*
BG 55	17-2	Spices	Kebab spice	*Bacillus safensis*
BG 56	18-1	Spices	Orange pepper	*Bacillus amyloliquefaciens*
BG 57	19-1	Fruits & vegetables	Apricots	*Bacillus mojavensis*
BG 58	20	Milk & dairy products	Livanjski Sir cheese	*Lysinibacillus fusiformis*
BG 59	21	Meat & meat products	Suho Meso (smoked dried beef)	*Bacillus paralicheniformis*
BG 60	41	Nuts, nut products & seeds	Pomegranade seeds	*Bacillus paralicheniformis*
BG 61	43	Nuts, nut products & seeds	Sunflower seeds	*Bacillus paralicheniformis*
BG 62	6; 56/1900	Milk & dairy products	Cheese smear	*Oceanobacillus oncorhynchi*
EGN 2	DSM 3007	Clinical isolate	Urine	*Acinetobacter baumanii*
EGN 3	NCTC 5866	Unknown	Unknown	*Acinetobacter iwoffii*
EGN 4	BF24	Clinical isolate	Blood culture	*Aeromonas caviae*
EGN 5	NCTC 8049	Milk & dairy products	Tin of milk with a fishy odor	*Aeromonas hydrophila*
EGN 7	CCM 4706	Clinical isolate	Rumen of bull	*Citrobacter amalonaticus*
EGN 8	DSM 30040	Clinical isolate	Faeces	*Citrobacter braakii*
EGN 9	NCTC 9750	Unknown	Unknown	*Citrobacter freundii*
EGN 11	CCM 2537	Clinical isolate	Blood culture	*Citrobacter koseri*
EGN 13	DSM 17578	Meat & meat products	Meat scraps	*Citrobacter youngae*
EGN 14	Cro1	Milk & dairy products	Milk powder	*Cronobacter sakazakii*
EGN 21	DSM 30053	Clinical isolate	Sputum	*Klebsiella aerogenes*
EGN 22	DSM 26481	Unknown	Unknown	*Enterobacter cloacae*
EGN 28	DSM 3493	Clinical isolate	Human knee lacteration	*Pantoea agglomerans*

EGN 31	ATCC 25922	Clinical isolate	Unknown	*Escherichia coli*
EGN 32	ATCC 35150	Clinical isolate	Human feces	*Escherichia coli*
EGN 33	ATCC 43895	Meat & meat products	Raw hamburger meat	*Escherichia coli*
EGN 34	DSM 30163	Unknown	Unknown	*Hafnia alvei*
EGN 35	NCTC 9528	Milk & dairy products	Butter	*Raoultella planticola*
EGN 36	DSMZ 5175	Clinical isolate	Pharyngeal tonsil	*Klebsiella oxytoca*
EGN 37	JCM 7251	Fruits & vegetables	Radish root	*Raoultella planticola*
EGN 39	DSM 6675	Clinical isolate	Stool of infant	*Morganella morganii*
EGN 40	DSM 4479	Unknown	Unknown	*Proteus mirabilis*
EGN 41	NCTC 10975	Clinical isolate	Human urine	*Proteus mirabilis*
EGN 42	NCTC 7475	Clinical isolate	Human faeces	*Providencia rettgeri*
EGN 43	ATCC 13315	Unknown	Unknown	*Proteus hauseri*
EGN 44	DSM 6676	Clinical isolate	Faeces of infant	*Providencia stuartii*
EGN 45	NCTC 10038	Environment	Freshwater pre-filter tanks	*Pseudomonas fluorescens*
EGN 46	NCTC 10662	Clinical isolate	Unknown	*Pseudomonas aeruginosa*
EGN 48	DSM 50342	Environment	Swimming-pool water	*Pseudomonas alcaligenes*
EGN 49	NCTC 10661	Environment	Forest soil	*Burkholderia cepacia*
EGN 50	DSM 1635	Unknown	Contaminant of *B. cereus* culture	*Brevundimonas dimenuta*
EGN 53	DSM 554	Unknown	Unknown	*Salmonella enterica*
EGN 54	NCTC 11935	Unknown	Unknown	*Serratia marcescens*
EGN 55	ATCC 8100	Unknown	Unknown	*Serratia marcescens*
EGN 56	ATCC 12022	Unknown	Unknown	*Shigella flexneri*
EGN 57	NCTC 8574	Unknown	Unknown	*Shigella sonnei*
EGN 64	DSM 18506	Fish & fishery products	Trout with red-mouth disease	*Yersinia ruckeri*
EGP 1	DSM 20124	Unknown	Unknown	*Arthrobacter globiformis*
EGP 2	DSM 20599	Meat & meat products	Bacon	*Brochothrix thermosphacta*
EGP 3	DSM 20109	Unknown	Unknown	*Cellulomonas flavigena*
EGP 4	CCM 1875	Unknown	Unknown	*Enterococcus faecalis*
EGP 5	CCM 4224	Clinical isolate	Urine	*Enterococcus faecalis*
EGP 6	CCM 3321	Environment	Milking machine	*Kurthia gibsonii*
EGP 7	DSM 20580	Clinical isolate	Turkey caecum	*Kurthia zopfii*
EGP 8	LMG 6907=BF17	Milk & dairy products	Raw milk	*Lactobacillus plantarum*
EGP 9	1A	Milk & dairy products	Raw milk	*Staphylococcus chromogenes*
EGP 10	2A	Milk & dairy products	Raw milk	*Staphylococcus cohnii*
EGP 11	17B	Milk & dairy products	Raw milk	*Staphylococcus haemolyticus*
EGP 12	7B	Milk & dairy products	Raw milk	*Staphylococcus intermedius*
EGP 13	24; BF46	Meat & meat products	Meat loaf	*Staphylococcus sciuri*
EGP 19	1.1.	Vegetarian meat substitutes	Vegetarian sausage	*Lactobacillus plantarum*
EGP 20	1	Vegetarian meat substitutes	Vegetarian sausage	*Lactobacillus sakei*
EGP 21	1,2	Environment	Cheese processing environment	*Leuconostoc carnosum*
EGP 22	6KMS	Vegetarian meat substitutes	Vegetarian sausage	*Listeria monocytogenes*
EGP 23	7,2	Vegetarian meat substitutes	Vegetarian sausage	*Pediococcus pentosaceus*
EGP 24	ATCC 25923	Clinical isolate	Unknown	*Staphylococcus aureus*
EGP 25	12 V/05	Milk & dairy products	Raw milk	*Staphylococcus aureus*
EGP 26	6B	Vegetarian meat substitutes	Vegetarian sausage	*Staphylococcus auricularis*
EGP 27	2,1	Milk & dairy products	Raw milk	*Staphylococcus carnosus*
EGP 30	6A	Milk & dairy products	Raw milk	*Staphylococcus epidermidis*
EGP 33	19 (105)	Fish & fishery products	Shrimps	*Staphylococcus saprophyticus*
EGP 35	Ocla 18 (6)	Milk & dairy products	Raw milk	*Micrococcus aloeverae*
EGP 36	CCM 2730	Clinical isolate	Human skin	*Staphylococcus warneri*
EGP 37	16 (96)	Fish & fishery products	Frozen fish	*Staphylococcus xylosus*
EGP 39	20B	Fish & fishery products	Fish	*Staphylococcus xylosus*
EGP 40	53/4 (7) orange	Milk a& dairy products	Cheese smear	*Microbacterium arborescens*

Supplementary Table S4A. Background information on naturally contaminated food samples.

Isolate ID	Producer	Category	Source
75	A	Tea	Vietnamese green tea
36	B	Fish & fishery products	Dried crayfish
35	C	Fish & fishery products	Dried lizardfish
37	D	Fish & fishery products	Dried salted anchovies
66	E	Fish & fishery products	Dried salted anchovies
32	E	Fruits & vegetables	Pandan leaves
38	E	Fruits & vegetables	Tamarind paste, moist
105	F	Spices	Coriander powder
33	F	Fruits & vegetables	Bean sprouts
40	F	Fruits & vegetables	Goji berries
34	G	Fruits & vegetables	Galanga root
41	G	Nuts, nut products & seeds	Pomegranate seeds
47	G	Nuts, nut products & seeds	Ground egusi

Background information on naturally contaminated food samples

Supplementary Table S4B. Background information on naturally contaminated milk samples.

Isolate ID	Producer	Sample	Processing	Husbandry
4	H	Cow milk	PM, NHOM	ORG, HAY
42	H	Cow milk	PM, NHOM	ORG, HAY
70	H	Cow milk	PM, NHOM	ORG, HAY
11-D	I	Cow milk	PM, NHOM	ORG
41	I	Cow milk	PM, NHOM	ORG
71	I	Cow milk	PM, NHOM	ORG
12	J	Cow milk	PM-ESL, MICROF	ORG
16	J	Cow milk	PM, NHOM	ORG
17	J	Cow milk	PM, NHOM	ORG
84	J	Cow milk	PM, NHOM	ORG
27	K	Bactofugate	RAW	CONV
64	K	Buttermilk	PM	ORG
85	K	Cow milk	PM-ESL, MICROF	ORG
139	K	Cow milk	HM-ESL	ORG
141	K	Cow milk	HM-ESL	ORG
73	L	Cow milk	PM-ESL, MICROF	CONV
74	L	Cow milk	PM-ESL, MICROF	CONV
75	L	Cow milk	PM-ESL, MICROF	CONV
2	M	Cow milk	PM-ESL, MICROF	ORG
13	M	Cow milk	PM-ESL, MICROF	ORG
15	M	Cow milk	PM-ESL, MICROF	ORG
76	M	Cow milk	PM-ESL, MICROF	ORG
120	M	Cow milk	HM-ESL	ORG
123	M	Cow milk	PM-ESL, MICROF	ORG
125	M	Cow milk	PM-ESL, MICROF	ORG
127	M	Cow milk	PM-ESL, MICROF	ORG
128	M	Cow milk	HM-ESL	ORG
129	M	Cow milk	PM-ESL, MICROF	ORG
130	M	Cow milk	HM-ESL	ORG
131	M	Cow milk	PM-ESL, MICROF	ORG
132	M	Cow milk	PM-ESL, MICROF	ORG
68	N	Cow milk	HM-ESL	ORG
77	N	Cow milk	PM-ESL, MICROF	ORG
72	O	Cow milk	PM, NHOM	ORG

6	P	Goat milk	HM-ESL	CONV
5	Q	Sheep milk	PM	CONV
45	Q	Goat milk	PM	CONV
78	Q	Sheep milk	PM	CONV
88	Q	Sheep milk	PM	CONV
100	Q	Sheep milk	PM	CONV
1	R	Sheep milk	HM-ESL	ORG
8	R	Sheep milk	HM-ESL	ORG
9	R	Goat milk	HM-ESL	ORG
11-A	R	Goat milk	HM-ESL	ORG
11-B	R	Goat milk	HM-ESL	ORG
11-C	R	Goat milk	HM-ESL	ORG
43	R	Goat milk	HM-ESL	ORG
44	R	Sheep milk	HM-ESL	ORG
65	R	Goat milk	HM-ESL	ORG
66	R	Sheep milk	HM-ESL	ORG
67	R	Sheep milk	HM-ESL	ORG

Abbreviations: CONV — conventional husbandry; silage feeding; ESL — extended shelf life; HAY — hay feeding; HM — high-pasteurized milk; MICROF — micro-filtrated; NHOM — non–homogenized milk; PM — pasteurized milk; ORG — organic; RAW — raw milk.

Supplementary Table S5. Accordance in the detection of *Bacillus cereus* group in naturally contaminated samples (n = 64) before and after freezing on selective agar media.

Sample ID	Producer	Sample	MYP	MYP	HI	BRI	CH	BA	Accordance
			BF	AF					
42	H	Cow PM	P	P	P	P	P	P	6
71	I	Cow PM	P	P	P	P	P	P	6
84	J	Cow PM	P	P	P	P	P	P	6
85	K	Cow PM-ESL	P	P	P	P	P	P	6
120; 123; 129; 131; 132	M	Cow HM-ESL; Cow PM-ESL	P	P	P	P	P	P	6
45	Q	Goat PM	P	P	P	P	P	P	6
78; 88; 100	Q	Sheep PM	P	P	P	P	P	P	6
44; 67	R	Sheep HM-ESL	P	P	P	P	P	P	6
12	J	Cow PM-ESL		P	P	P	P	P	5
139	K	Cow HM-ESL		P	P	P	P	P	5
125; 128	M	Cow HM-ESL; Cow PM-ESL	P	P	P	P	P		5
43	R	Goat HM-ESL	P	P	P	P	P		5
65	R	Goat-HM-ESL	P	P	P	P		P	5
66	R	Sheep HM-ESL	P	P	P	P	P		5
36	B	Dried crayfish	P	P			P	P	4
40	F	Goji berries		P		P	P	P	4
34	G	Galanga root		P		P	P	P	4
4	H	Cow PM	P	P	P		P		4
141	K	Cow HM-ESL		P	P	P	P		4
6	P	Goat HM-ESL		P		P	P	P	4
5	Q	Sheep PM			P	P	P	P	4
75	A	Vietnamese green tea	P		P			P	3
37	D	Dried salted anchovies				P	P	P	3
33	F	Bean sprouts				P	P	P	3
41	G	Pomegranate seeds		P			P	P	3
17	J	Cow PM		P		P	P		3
13	M	Cow PM-ESL			P	P		P	3
72	O	Cow PM	P	P	P				3
11-C	R	Goat HM-ESL				P	P	P	3
35	C	Dried lizard fish	P					P	2
66	E	Dried salted anchovies	P					P	2
47	G	Ground egusi	P					P	2
16	J	Cow PM				P	P		2
15	M	Cow PM-ESL	P			P			2
127; 130	M	Cow PM-ESL	P	P					2
1	R	Sheep HM-ESL	P					P	2
8; 9	R	Sheep HM-ESL; Goat HM-ESL			P			P	2
11-A	R	Goat HM-ESL	P					P	2

32	E	Pandan leaves					P		1
32	E	Pandan leaves					P		1
38	E	Tamarind paste		P					1
105	F	Coriander powder	P						1
70	H	Cow PM	P						1
11-D	I	Cow PM					P		1
41	I	Cow PM	P						1
27; 64	K	Bactofugate RM; Butter milk	P						1
73; 74; 75	L	Cow PM-ESL	P						1
2	M	Cow PM-ESL						P	1
76	M	Cow PM-ESL	P						1
68; 77	N	Cow HM-ESL; Cow PM-ESL	P						1
11-B	R	Goat HM-ESL		P					1

Abbreviations: AF — after freezing; BA — BACARA® agar; BF — before freezing; BRI — Brilliance™ *B. cereus* agar; CH — CHROMagar™ *B. cereus*; HI — ChromoSelect *Bacillus* agar; HM — high-pasteurized/high heat-treated milk; MYP — mannitol egg yolk polymyxin agar; PM — pasteurized milk; RM — raw/not heat-treated milk; Yellow color — before and after freezing positive on MYP agar; Green color — before freezing positive on MYP agar; Blue color— after freezing positive on MYP agar; Grey color-after freezing positive on chromogenic media.

Supplementary Table S6. *panC* group and toxin gene profile combinations of *Bacillus cereus* group detected before and after freezing (*n* = 64 samples).

**Sample**	**Producer**	**BF**					**AF**						
Vietnamese green tea	A			IV/A						IV/A			
Dried crayfish	B		III/F					III/A	III/C				
Dried lizard fish	C		III/F					III/A	III/F				
Dried salted anchovies	D									IV/A			VI/C
Dried salted anchovies	E		III/D						III/D				
Pandan leaves	E								III/F				
Tamarind paste	E								III/F				
Coriander powder	F			IV/A									
Bean sprouts	F									IV/A	IV/D		
Goji berries	F									IV/A	IV/D		
Pomegranate seeds	G						II/C						
Ground egusi	G		III/D						III/D				
Galanga root	G								III/F		IV/D		
Cow PM	H						II/C			IV/A			
Cow PM	H	II/F		IV/A		VI/A				IV/A			VI/C
Cow PM	H	II/F				VI/C							
Cow PM	I									IV/A			
Cow PM	I					VI/C							
Cow PM	I					VI/C							VI/C
Cow PM-ESL	J						II/F			IV/A			
Cow PM	J						II/F			IV/A			
Cow PM	J												VI/C
Cow PM	J			IV/A						IV/A			
Bactofugate RM	K		III/F										
Butter milk	K		III/F										
Cow PM-ESL	K			IV/A									VI/C
Cow HM-ESL	K									IV/A			
Cow HM-ESL	K									IV/A			
Cow PM-ESL	L	II/C											
Cow PM-ESL	L					VI/C							
Cow PM-ESL	L			IV/A									
Cow PM-ESL	M									IV/A			
Cow PM-ESL	M									IV/A			
Cow PM-ESL	M					VI/C							VI/C
Cow PM-ESL	M					VI/A							
Cow HM-ESL	M		III/F							IV/A			
Cow-PM-ESL	M		III/F						III/F				
Cow-PM-ESL	M			IV/A						IV/A			
Cow PM-ESL	M		III/D										VI/C

Cow HM-ESL	M			IV/A								V/C	
Cow PM-ESL	M			IV/A						IV/A			
Cow HM-ESL	M				V/C					IV/A			
Cow PM-ESL	M		III/F						III/F				
Cow PM-ESL	M		III/F						III/F				
Cow HM-ESL	N	II/F											
Cow PM-ESL	N	II/C											
Cow PM	O	II/C		IV/A		VI/F	II/F						VI/F
Goat HM-ESL	P								III/F	IV/A			
Sheep PM	Q								III/F	IV/A			
Goat PM	Q		III/F			VI/A			III/F	IV/A			
Sheep PM	Q					VI/A			III/F	IV/A			
Sheep PM	Q					VI/C			III/F	IV/A			
Sheep PM	Q		III/F						III/F				
Sheep HM-ESL	R	II/C					II/C						
Sheep HM-ESL	R								III/F	IV/A			
Goat HM-ESL	R								III/F	IV/A			
Goat HM-ESL	R					VI/A	II/F			IV/A			
Sheep HM-ESL	R					VI/A			III/F	IV/A			
Goat HM-ESL	R	II/F		IV/A			II/F		III/F	IV/A			
Sheep HM-ESL	R	II/F					II/F		III/F	IV/A			
Sheep HM-ESL	R	II/F								IV/A			
Goat HM-ESL	R	II/C											
Goat HM-ESL	R						II/C						
Goat HM-ESL	R						II/C		III/F				

Abbreviations: A– toxin profile A (*nhe*+, *hbl*+, *cytK*+); AF – after freezing, BF – before freezing; C – toxin profile C (*nhe*+, *hbl*+); D – toxin profile D (*nhe*+, *cytK*+); ESL – extended shelf life; F – toxin profile F (*nhe*+); HM – high-pasteurized milk; PM – pasteurized milk; RM – raw milk; II – *panC* group II (cytotoxic, growth ≥ 7°C); III – *panC* group III (cytotoxic-highly cytotoxic, growth ≥ 15°C); IV – *panC* group IV (cytotoxic-highly cytotoxic, growth ≥ 10°C); V – *panC* group V (low cytotoxic, growth ≥ 8°C); VI – *panC* group VI (non or low cytotoxic; growth ≥5°C); Italic – detected before and after freezing. The different color coding indicates different *panC*/toxin profile combination types.
